# Nano zinc oxide – An alternate zinc supplement for livestock

**DOI:** 10.14202/vetworld.2020.121-126

**Published:** 2020-01-16

**Authors:** K. Geetha, M. Chellapandian, N. Arulnathan, A. Ramanathan

**Affiliations:** 1 Nanotechnology Division, Periyar Maniammai Institute of Science and Technology, Thanjavur, Tamil Nadu, India; 2 Department of Animal Nutrition, Veterinary College and Research Institute, Tirunelveli, Tamil Nadu, India; 3 Department of Animal Husbandry, School of Agriculture and Animal Sciences, Gandhigram Rural Institute, Dindigul, Tamil Nadu, India

**Keywords:** antimicrobial, cytotoxicity, nano zinc oxide, precipitation method, zinc supplementation

## Abstract

**Aim:**

This study was aimed to investigate antimicrobial and cytotoxicity effect of nano ZnO in *in vitro* for the application of livestock feed supplement.

**Materials and Methods:**

Nano ZnO was synthesized by wet chemical precipitation method using zinc acetate as a precursor and sodium hydroxide was used for reducing the precursor salt. The properties of synthesized powder were characterized using ultraviolet (UV)–visible spectroscopy, Fourier transform infrared (FTIR), scanning electron microscopy (SEM), and X-ray diffraction (XRD), respectively. *In vitro* antimicrobial activities were analyzed against the pathogenic bacteria in poultry *Escherichia coli*, *Staphylococcus aureus*, *Klebsiella pneumoniae*, and *Streptococcus aeruginosa*. 3-(4,5-dimethylthiazol-2-yl)-2,5-diphenyltetrazolium bromide assay was conducted to analyze the cytotoxicity effect of nano ZnO.

**Results:**

SEM showed a spherical ZnO particle in the range of 70-100 nm. The size of the particle and purity of the sample were confirmed by XRD. The nano-sized ZnO particles exhibited the UV absorption peak at 335 nm. In FTIR spectroscopy, pure ZnO nanoparticles showed stretching vibrations at 4000-5000 cm^−1^. ZnO nanoparticles exhibited remarkable antibacterial activity against *E. coli*, *S. aureus*, *K. pneumoniae*, and *S. aeruginosa* bacterial strains. Cell viability was significantly reduced in a dose-dependent manner in the cytotoxicity study.

**Conclusion:**

From the broad-spectrum antibacterial activity and the lower cytotoxicity observed at the prescribed dose, it is concluded that nano ZnO powder is a potential alternate zinc supplement for livestock.

## Introduction

Zinc is the second most essential trace element in all living systems from animals to humans, plays an essential role in many metabolic processes of the body [1]. The daily dietary intake of zinc is essential to regulate the cell division by regulating the synthesis of protein and DNA [2]. The two predominant sources of Zn used by the animal feed industry are ZnO and ZnSO_4_.H_2_O [3]. Deficiency of zinc in cattle leads to improper growth, reduced feed intake, reduced milk yield, and decreases of cycling and conception rate [4,5]. Milk yield increased when Zn is supplemented in the form of zinc methionine or zinc lysine to the cattle [6]. The National Research Council recommended 30 ppm (mg/kg) as the dietary requirement of zinc on a dry matter basis for cattle. Supplementation of nano zinc drastically reduced somatic cell count in milk from cows with subclinical mastitis and improved milk production than cows supplemented with macro zinc oxide [7,8]. Zinc deficiency in lamb results in slipping of wool, decreased growth and improper growth of testes [9], weight loss during lactation, development of skin lesions, and excessive salivation [10]. Elevation of phytate by poor intestinal absorption of zinc from improper zinc supplement ends in prolonged enteritis and dermatosis [11]. Continuous supplementation of zinc in the form of zinc sulfate (10 mg/kg/day) or zinc methionate (1.7 mg/kg/day) is normally required for maintenance [12]. Zinc plays with disease resistance, cellular immunity, spleen development, and alteration in high-density lipoprotein cholesterol in poultry [13,14]. The supplemental zinc used in poultry is zinc sulfate or zinc chloride [15]. Zinc in the form of Zn methionine shows greater biological availability than zinc from inorganic sources [16]. The recommended level of zinc in various poultry diets ranges from 40 to 75 ppm [17].

Zinc oxide is the most commonly used zinc supplement with high antibacterial activity, antifungal, and growth promoter ability [18]. Zinc oxide generates hydrogen peroxide which can pass through the cell wall, disrupt metabolic process, and, in turn, inhibit the microbial growth. The affinity of zinc oxide toward the bacterial cell is the most important factor for antibacterial activity [19]. It reduces zinc deficiency and its results to reduce growth retardation and lower rate of infertility [20]. However, the bioavailability of ZnO can be enhanced by changing the size effect. The reduced size of ZnO in nanoscale will enhance the bioavailability by increased ionization of zinc. Commonly organic zinc resources are resulted with good results due to the higher bioavailability in all livestock growth and production. However, the production cost and supplementation dose rate are not sufficient to make artificial farming in a cost-effective manner. The nano ZnO can produce a positive effect to overcome the zinc deficiency problem with cost-effective and lower dose rate.

We hypothesized that the higher bioavailability of nano ZnO can easily absorb from the intestine and interferes with subcellular mechanisms. Moreover, the highest antibacterial effect with different bacterial species was reported in recent works. Nano ZnO in feed mixture will provide the dual function of a Zn supplement and as an antimicrobial agent during feed storage. To test this supposition, we have synthesized the nano ZnO by wet chemical precipitation method at 80°C using zinc acetate. The confirmation of nano ZnO presence, concentration, morphology, and particle size is characterized using ultraviolet (UV)–visible spectroscopy, Fourier transform infrared (FTIR), scanning electron microscopy (SEM), and X-ray diffraction (XRD), respectively. Antimicrobial effect against *Escherichia coli*, *Staphylococcus aureus*, *Klebsiella pneumoniae*, and *Streptococcus aeruginosa* in *in vitro* level was evaluated by antibacterial disk diffusion test. MTT cytotoxicity assay was carried out to determine the biocompatibility and cytotoxic effect of nano ZnO.

## Materials and Methods

### Ethical approval

No Ethical Committee approval was necessary for this study as we conducted experiment *in vitro*.

### Precipitation method

The zinc oxide nanoparticles were synthesized using zinc acetate as the precursor and sodium hydroxide as the reducing agent. A 0.1 M of zinc acetate homogenous mixture was dissolved in double-distilled water at pH of 11 for 2 h with the aid of magnetic stirrer. The 0.1 M NaOH solution was slowly added into the zinc acetate solution under continuous stirring. The final solution was stirred for 4 h at pH of 7. The final precipitate was filtered with Whatman No.1 filter paper and then the colloidal zinc oxide was lyophilized. Then, the powdered zinc oxide nanoparticles were collected and stored for further process.

Zn(CH_3_COO)_2_ + 2NaOH→Zn(OH)_2_ + 2CH_3_COONa

Zn(OH)_2_→ZnO + H_2_O

### Characterization

The obtained samples were characterized by powder XRD method with CuKα X-ray radiation (λ=0.15496 nm). The surface morphology of the sample was revealed by SEM (TESCAN, VEGA3 LMU). The composition of the elements was analyzed by the use of FTIR spectroscopy (PerkinElmer Spectrum RX I) and the optical absorption spectrum of nano ZnO powder was derived from UV–visible spectroscopy (UV 1800 spectrophotometer, SHIMADZU).

### Antimicrobial activity

The bacterial strains such as *E. coli*, *S. aureus*, *K. pneumoniae*, and *S. aeruginosa* were purchased from Microbial Type Culture Collection, Chandigarh, India. Actively growing test bacterial strains were spread on four wells made in the nutrient agar plate. The zinc oxide solution with different concentrations such as 50, 100, and 150 µg/ml was loaded in each well, while one well was filled with only broth medium as a control. Then, the plates were incubated at 37°C for 24-48 h. Antimicrobial activity was expressed as a diameter (mm) of the inhibitory zone.

### Cytotoxicity assay

The cytotoxicity assay of the prepared ZnO nanoparticle was measured using MTT test. The mouse fibroblast (L-929) at a density of 1×10^6^ cellll was pipetted into tissue culture with 12 wells, allowed to incubate for 24 h and treated with different concentrations (50-500 µl/ml) of ZnO nanoparticles. After the ZnO nanoparticle treatment, the medium was changed and the cells were washed twice with (Dulbecco’s Modified Eagle’s Medium/Ham’s 12 nutrient mixtures) without fetal calf serum to remove the dead cells. The cells were incubated with 200 µl (5 mg/ml) of MTT reagent for 6-7 h at 37°C in 5% CO_2_ incubator for cytotoxicity. Tetrazolium salt MTT converted to a colored formazan by the mitochondrial dehydrogenases indicates the presence of viable cells. Color development was measured at 595 nm using a spectrophotometer. In this assay, cells without nanoparticle attachment were used as a control. The viability of the cell was calculated as follows:


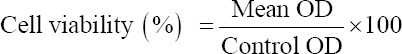


## Results and Discussion

### SEM analysis

**Figure-1:**
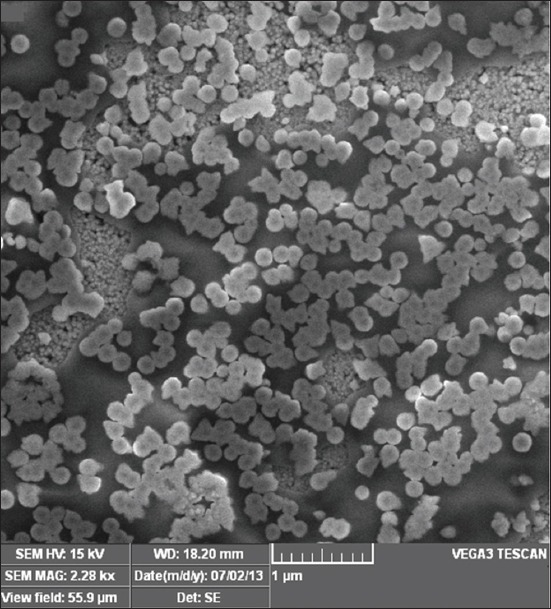
Scanning electron microscopy image of ZnO nanoparticles.

The SEM photograph of the sample is shown in Figure-1. The SEM images of ZnO samples obtained from the precipitation method revealed the presence of nanoparticles of spherical shape with minimal agglomeration. A similar structure was observed in ZnO nanoparticles by Kim and Park [21], Ong *et al*. [22]. The capping agent might be used to reduce the particle size during precipitation. The particle size varied from 70 to 100 nm as observed from the SEM image shown below. Reducing the rate of addition of sodium hydroxide with zinc acetate might reduce the particle size formation during precipitation.

### XRD studies

**Figure-2:**
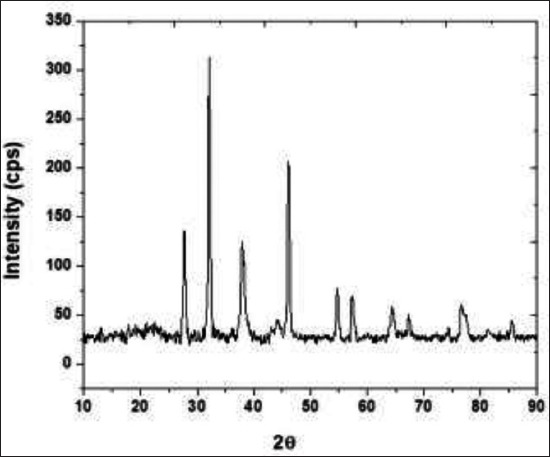
Spectrum of ZnO nanoparticles obtained by X-ray diffraction spectroscopy.

Figure-2 shows the XRD patterns of ZnO samples. Bragg reflection with 2θ of A 32.18°, 36.78°, and 47.54° was observed to (100), (101), and (102) planes confirm the presence of ZnO nanoparticle. Furthermore, the less intense peaks at 48°, 54°, 57°, 64°, and 77° (2θ values) indicate the high crystallinity of ZnO samples and high purity of the ZnO nanopowders. Crystallite size of the ZnO samples was calculated using Scherrer’s formula. The average particle size of the sample obtained from this precipitation method was calculated using full width at half maximum of more intense peak corresponding to 101 planes located at 32.18° using Scherrer’s formula. The average crystalline size is found to be 74.67 nm. Similarly, XRD pattern was reported by Kim and Park [21], Mohana and Renjanadevi [23], and Jenkins and Snyder [24].

### FTIR spectra

**Figure-3:**
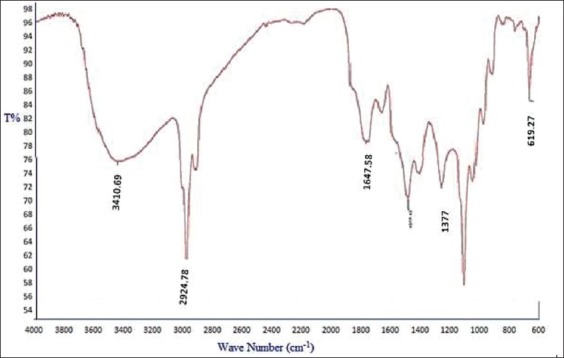
Fourier transform infrared spectra of ZnO nanoparticles.

FTIR spectrum of the synthesized ZnO nanoparticles showed (Figure-3) the fundamental mode of vibration at 3410.69 which corresponds to the O-H stretching vibration, 2924.78 which corresponds to C-H stretching vibration, and 1377.13 corresponds to C=O asymmetric stretching vibration. The peaks 1647.58 and 619.27 correspond to ZnO stretching and deformation vibration. The absorption at 857 cm^−1^ is due to the formation of tetrahedral coordination of Zn. The frequencies observed for the zinc oxides are in accordance with literature values [25-27] reported similar FTIR spectra of zinc oxide nanoparticles in their investigation.

### UV–visible absorption spectrum

**Figure-4:**
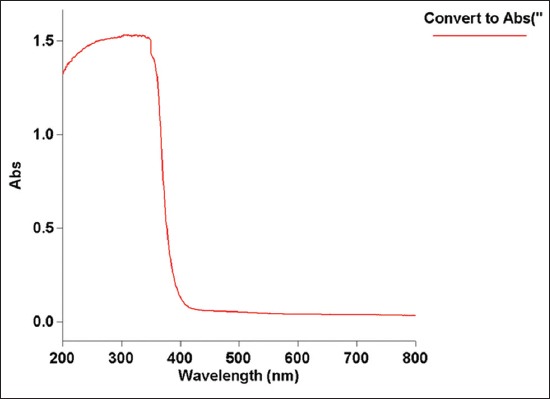
Ultraviolet–visible absorption of the ZnO nanoparticles.

UV–visible absorption spectroscopy is a commonly used technique to examine the optical properties of nanosized particles. It is obvious from Figure-4, nano zinc oxide powder exhibits a strong absorption band at about 335 nm, which lies below the bandgap wavelength of 388 nm of bulk ZnO. The excitation absorption of ZnO powder and bulk ZnO material appeared at ~327 nm and ~373 nm was reported. The excitation peak at 335 in Figure-4 is similar to the previous report [28,29].

### Antibacterial activity

The antibacterial activity of control along with ZnO nanoparticles was investigated against pathogenic bacteria such as *E. coli*, *S. aureus*, *K. pneumoniae*, and *S. aeruginosa*. Table-1 pronounces the ZnO exhibited remarkable antibacterial activity against tested bacterial strains. It has already been proved that nano-sized ZnO suspensions are active in inhibiting bacterial growth. In the present study, ZnO nanoparticle was found to have a broad spectrum of antibacterial activity. A significant inhibitory effect rate was observed against the selected bacteria in the order of *E. coli*, *S. aureus*, *K. pneumoniae*, and *S. aeruginosa*. It seems that active oxygen species generated by ZnO nanoparticles could be responsible for the antimicrobial activity. Antibacterial activity of nano ZnO against *E. coli* has been reported and the reactive oxygen species induced by the nano ZnO are responsible for inhibiting bacterial growth [20,30].

**Table-1:** Antimicrobial activity of nano ZnO.

Bacteria	Concentration (µg/ml)	Zone of inhibition by ZnO nanoparticles (mm)
*Staphylococcus aureus*	50	14
	100	17
	150	19
*Escherichia coli*	50	11
	100	19
	150	24
*Klebsiella pneumoniae*	50	07
	100	11
	150	14
*Streptococcus aeruginosa*	50	12
	100	15
	150	17

### Cytotoxicity assay

**Figure-5:**
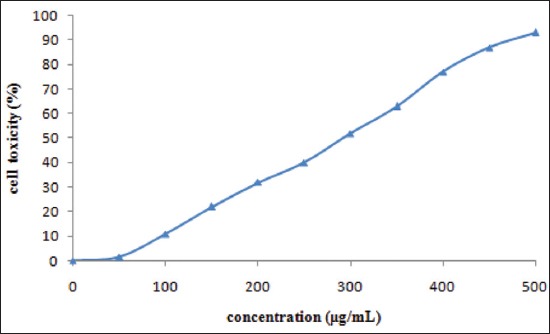
Cytotoxicity of ZnO nanoparticles on L-929 (mouse fibroblast) cell line.

The cytotoxic effect of ZnO nanoparticle was determined using mouse epithelial cell L-929 by MTT assay. The cytotoxicity rate was increased with increased concentration of nano ZnO (Figure-5). A significant cytotoxic effect started from a concentration of 180 µg/ml, whereas up to 180 µg/ml, the minimal acceptable toxicity level 30% was observed. Hence, the ZnO nanoparticle can be used as a feed supplement at a dose rate of up to 180 µg/ml.

## Conclusion

The larger particle ZnO is not commonly used in livestock feed supplementation due to its low bioavailability. However, the nanoparticulated ZnO can provide a better surface to volume ratio for the physiological digestive mechanism of zinc. Hence, the bioavailability of the zinc might be enhanced by nano ZnO supplementation than larger particulate ZnO or zinc methionine supplements. In this context, the ZnO nanoparticles were synthesized by precipitation method using zinc acetate. SEM analyses revealed that the synthesized ZnO was spherical in shape with a diameter of 70-100 nm. The same size and purity of the sample are revealed by XRD. The nano-sized ZnO particles exhibited the UV absorption peak at 335 nm. In FTIR spectroscopy, pure ZnO nanoparticles showed stretching vibrations at 4000-5000 cm^−1^. The antibacterial test proved that the prepared ZnO can resist the growth of tested bacteria. The cell cytotoxicity study expressed that the lethal dose is only above 180 µg/ml. Hence, the antibacterial activity (150 µg/ml) and the cell viability dose level (up to 180 µg/ml) are unique in the conducted experiment. Hence, it is proposed to conduct the feeding trails in animals with ZnO nanoparticle to assess their feeding value.

## Authors’ Contributions

KG designed the experiment, synthesized, and characterized the nano ZnO. NA helped in antimicrobial and MTT assay. KG drafted the manuscript. MC helped in drafting of the manuscript. MC and AR reviewed and corrected the manuscript. All authors read and approved the final manuscript.
